# Inhibition of EZH2 prevents acute respiratory distress syndrome (ARDS)-associated pulmonary fibrosis by regulating the macrophage polarization phenotype

**DOI:** 10.1186/s12931-021-01785-x

**Published:** 2021-07-03

**Authors:** Xiaowei Bao, Xiandong Liu, Na Liu, Shougang Zhuang, Qian Yang, Huijuan Ren, Dongyang Zhao, Jianwen Bai, Xiaohui Zhou, Lunxian Tang

**Affiliations:** 1 Department of Internal Emergency Medicine and Critical Care, Shanghai East Hospital, Tong Ji University, 1800, Yuntai Road, Shanghai, 200120 China; 2 Department of Nephrology, Shanghai East Hospital, Tongji University School of Medicine, Shanghai, China; 3 Department of Medicine, Rhode Island Hospital and Alpert Medical School, Brown University, Providence, RI USA; 4 Research Center for Translational Medicine, Shanghai East Hospital, Tongji University, 150, Jimo Road, Shanghai, 200120 China

**Keywords:** ARDS, Sepsis, Fibrosis, EZH2, Macrophage polarization, EMT

## Abstract

**Background:**

We recently reported histone methyltransferase enhancer of zeste homolog 2 (EZH2) as a key epigenetic regulator that contributes to the dysfunction of innate immune responses to sepsis and subsequent lung injury by mediating the imbalance of macrophage polarization. However, the role of EZH2 in acute respiratory distress syndrome (ARDS)-associated fibrosis remains poorly understood.

**Methods:**

In this study, we investigated the role and mechanisms of EZH2 in pulmonary fibrosis in a murine model of LPS-induced ARDS and in ex-vivo cultured alveolar macrophages (MH-S) and mouse lung epithelial cell line (MLE-12) by using 3-deazaneplanocin A (3-DZNeP) and EZH2 the small interfering (si) RNA.

**Results:**

We found that treatment with 3-DZNeP significantly ameliorated the LPS-induced direct lung injury and fibroproliferation by blocking EMT through TGF-β1/Smad signaling pathway and regulating shift of macrophage phenotypes. In the ex-vivo polarized alveolar macrophages cells, treatment with EZH2 siRNA or 3-DZNeP suppressed the M1 while promoted the M2 macrophage differentiation through modulating the STAT/SOCS signaling pathway and activating PPAR-γ. Moreover, we identified that blockade of EZH2 with 3-DZNeP suppressed the epithelial to mesenchymal transition (EMT) in co-cultured bronchoalveolar lavage fluid (BALF) and mouse lung epithelial cell line through down-regulation of TGF-β1, TGF-βR1, Smad2 while up-regulation of Smad7 expression.

**Conclusions:**

These results indicate that EZH2 is involved in the pathological process of ARDS-associated pulmonary fibrosis. Targeting EZH2 may be a potential therapeutic strategy to prevent and treat pulmonary fibrosis post ARDS.

## Background

Acute respiratory distress syndrome (ARDS) remains a major clinical challenge in critically ill patients which represents a stereotypic response to lung injury with transition from exudative inflammatory responses to a fibroproliferative phase [1]. Most patients with ARDS survive the acute phase, but many succumb to death, often in association with pulmonary fibrosis [1, 2]. Progressive pulmonary fibrosis has become the major cause of mortality in ARDS patients, however no pharmacological treatment has so far shown efficacy to ameliorate the prognosis [1, 2].

Despite increased understanding of the pathogenesis of ARDS, the underlying mechanism of the balance between resolution of injury and inappropriate or ineffective tissue repair is complex and still needs to be elucidated. Apart from alveolar fibroblasts and epithelial cells, macrophages are also essential for the rehabilitation and fibrotic phase of acute lung injury (ALI)/ARDS [3]. Previous studies have indicated that after pathogenic factors are eliminated, resident and recruited macrophages are still able to shift from the M1 subtype to the anti-inflammatory M2 phenotype, a central driver for the resolution of lung inflammation and subsequent fibrosis by regulating extracellular matrix turnover [3, 4]. We have previously reported that ALI mice treated with *ex-vivo* programmed M2a or M2c macrophages showed a reduction of lung fibrosis [5], and Tim3 + Treg had the ability to resolve the inflammation and fibrosis of ALI by inducing M2-like macrophages [6]. Although the mechanisms of the phenotypic change from the M1 to the M2 macrophage have been extensively studied, the epigenetic modification of this process is only beginning to emerge. A growing body of research suggests that histone modifications are major regulators of macrophage functions [7]. In particular, EZH2 is the catalytic component of the polycomb-repressive complex 2 (PRC2) which catalyzes the trimethylation of histone H3 at lysine 27 (H3K27me3) and has been suggested to play key roles in regulating immune cell functions [8]. A recent study has documented that EZH2 deficiency attenuated the inflammatory responses in macrophages, leading to the suppression of autoimmune inflammation [9]. Using a cecal ligation and puncture-induced ALI mice model, we further demonstrated that pharmacological inhibition of EZH2 suppressed the M1 polarization in vivo and had the tendency to promote the M2 subtype through the SOCS3/STAT1 pathway [10]. Numerous studies have indicated that EZH2 appears to promote fibrosis. Its expression is increased in patients with idiopathic pulmonary fibrosis [11] and chronic kidney disease [12], as well as in animal models including bleomycin-induced lung fibrosis [11], carbon tetrachloride liver fibrosis [13], unilateral ureteral obstruction kidney fibrosis [12] and peritoneal fibrosis [14]. Currently, pharmacological inhibition of EZH2 activity has become a new strategy for anti-tumor and anti-fibrosis therapy in preclinical studies [15]. 3-deazaneplanocin A (3-DZNeP) is an inhibitor of S-adenosylhomocysteine (SAH) hydrolase, which can inhibit the methylation level of H3K27 and the activity of EZH2 [16]. Recently it has been reported 3-DZNeP can effectively prevent tumor progression and fibrosis [15, 16]. However, it remains unknown whether EZH2 is involved in ARDS-associated pulmonary fibrosis.

In this study, we examined the effect of the EZH2 inhibitor on LPS-induced pulmonary fibrosis in mice and explored the possible mechanism involved. Furthermore, we evaluated the pharmacological and genetic inhibition of EZH2 on macrophage functions under different polarization states.

## Methods

### Animal models and experimental design

All the experiments were performed in accordance with the animal experimentation guidelines of Tongji University School of Medicine, PR China. Male C57BL/6 mice (Shanghai Super-B&K Laboratory Animal Corp. Ltd., Shanghai, PR China) aged 6–8 weeks (16–18 g) were housed in a pathogen-free facility at Tongji University. Mice were anesthetized with intraperitoneal ketamine and acetypromazine (150 and 13.5 mg/kg respectively) before tracheal exposure [5, 6]. LPS (5 μg/g, *Escherichia coli* O55:B5; sigma) dissolved in 100 μl phosphate buffered solution (PBS) was instilled intratracheally via 20-gauge catheter as we previously reported [5, 6]. A total of 240 animals were randomly assigned to the following four groups (N = 15/group) at each time points (Day1, Day 3, Day 7 and Day 14) after the administering of LPS: (a) Control animals (Control) were instilled intratracheally with 100 μl PBS without LPS (Con group); (b) Control + 3-DZNeP animals received intra-peritoneal 3-DZNeP dissolved in DMSO (50 mg/kg) 24 h before and 1 h after PBS instillation (Con + 3-DZNeP); (c) Vehicle treated ALI animals received intra-peritoneal vehicle DMSO 24 h before and 1 h after LPS instillation (ALI group) and (d) 3-DZNeP treated animals received intra-peritoneal 3-DZNeP dissolved in DMSO (50 mg/kg) 24 h before and 1 h after LPS instillation (ALI + 3-DZNeP group). All the mice survived for 14 days in the control and control + 3-DZNep groups. For the ALI group, 1 mouse was dead at Day 3, 5 mice were dead at Day 7 and 6 mice were dead at Day 14 separately. While we only noticed 2 mice were dead at Day 7 and 3 mice were dead at Day 14 in the ALI + 3-DZNeP groups. Bronchoalveolar lavage fluid (BALF) sample and lung tissue were obtained at Day 1, Day 3, Day 7 and Day 14 as our previous description [5, 6]. Analysis of BALF was conducted and wet/dry weight ratio was calculated as we previously described [5, 6].

### Masson trichrome staining and hydroxyproline assay

Left lower lobe inflated to 25 cm H_2_O with 1% low-melting agarose (Invitrogen, Grand Island, NY) for the evaluation by Masson trichrome according to the procedure described in our previous study [5, 6]. Using Image pro-Plus 6.0 Software (Media-Cybernetics, Silver Spring, Md), the collagen tissue area was measured by drawing a line around the perimeter of the positive staining area, and calculating and graphing the average ratio to each microscopic field (200).

Pulmonary collagen secretion and deposition in ALI mice were quantitatively analyzed by using the hydroxyproline assay according to the procedure as we previously described [5, 6]. Results were expressed as micrograms of hydroxyproline.

### Isolation of BALF macrophages and co-culture experiments

According to the previous report [17], BALF harvesting from the above mentioned animal groups was filtered through gauze sponge (USP, type VII 199 Gauze, 12 Ply) to remove debris and cells were pelleted by centrifugation at 275×*g* for 10 min. After lysis of red blood cells with ACK buffer (#118–156-101; Quality Biological, Gaithersburg, 201 MD), BALF cells were pelleted by centrifugation for 5 min at 275×*g* and resuspended in RPMI-1640 media containing 10% fetal bovine serum (FBS) at a concentration of 1 × 10^5^ macrophages per well for 96-well plate experiments. Macrophages were adhered to plastic tissue culture plates for 2 h and non adherent cells were removed by washes with RPMI-1640 for 96-well plate. To analyze purity of macrophages, the resulting cells were stained with fluorescein isothiocyanate (FITC)-conjugated anti-mouse CD19 (B cell markers), CD49b (NK marker), phycoerythrin (PE)-conjugated antimouse CD11b (myeloid cell marker), and allophycocyanin-conjugated anti-mouse CD11c (dendritic marker) and were subsequently analyzed using a flow cytometer, which yielded a purity of > 95% macrophages.

Mouse lung epithelial cell line (MLE-12, ATCC, US) and the above isolated BALF macrophages were co-cultured using Transwells (3422, Corning, MA, US) with a membrane pore size of 8 μm. MLE-12 cells were seeded into upper 24-well plates at a density of 6 × 10^3^ cells per wells, and the BALF macrophages from different groups were seeded into the lower chamber. 48 h later, cells and supernatants were harvested for subsequent examination.

### Cell culture and treatments

MH-S cells, the SV40 transformed mouse alveolar macrophage cell line (CRI-2019, ATCC, Baltimore, Md, USA), were cultured in RPMI-1640 medium (Gibco, CA, USA) containing 10% fetal bovine serum (Gibco, Australia Origin) and 1% penicillin/streptomycin in an atmosphere of 5% CO2, and 95% air at 37℃. According to the previous report [18], MH-S cells were polarized as M1 or M2 macrophages with the indicated stimulants: 50 ng/mL LPS (L3024, Sigma, Mo) 24 h for M1 polarization, and 10 ng/mL IL-4 (404-ML, R&D Systems) 24 h for M2 polarization. The 3-DZNeP (Selleckchem, Houston, TX, USA) (0, 1, 2, 5 and 10 μM) was added 24 h before LPS or IL-4 stimulation. The small interfering (si) RNA oligonucleotides targeted to EZH2 (GenePharma, Shanghai, PR China) were used to knock down EZH2 more specifically in vitro. The MH-S cells were transfected with EZH2 siRNA (100 pmol) with lipofectamine 2000 (Invitrogen, Grand Island, NY, USA) or scrambled siRNA (100 pmol) according to the manufacturer’s instructions 24 h before LPS or IL-4 stimulation. After different stimulations, cells and supernatants were harvested for analyses. All of the in vitro experiments were repeated at least three times.

### Cell viability and proliferation assays

The in vitro viability and proliferation of the 3-DZNeP-treated MH-S cells was examined in Trypan-Blue-exclusion and 3, 4, 5-(dimethylthiazol-2-yl) 2, 5-diphenyl tetrazolium bromide (MTT) assays as previously described [19]. Briefly, MH-S cells were cultured in 96-well culture plates (200 μl/well) in DMEM containing 10% FBS in the various concentrations of 3-DZNeP (0, 1, 2, 5 and 10 μM) for 48 h at 37 °C at 5% CO2. The cells were manually counted under the phase-contrast microscope by using a hemocytometer. Effects of 3-DZNeP on MH-S cell proliferation were determined in MTT assays according to the manufacturer’s instructions (Cell Proliferation Assay kit, Promega).

### ELISA analyses

ELISAs were performed to measure concentrations of TNF-α, IL-1β, TGF-β1, IL-6 and IL-10 protein on supernatants of lung homogenates, BALF and cell culture supernatants, which was performed in accordance with the manufacturer’s instructions (R&D Systems).

### Immunoblotting analysis

Immunoblotting analysis was conducted as described previously [5, 6]. Densitometry analysis of immunoblot results was conducted by using ImageJ software. Data are expressed as mean ± SEM of three replicated experiments (Primary antibodies are listed in supplementary material 1, Table S1).

### Real-time PCR (qRT-PCR)

All reagents, primers, and probes were obtained from Applied Biosystems. A GAPDH endogenous control was used for normalization. Reverse transcriptase reactions and real-time PCR were performed according to the manufacturer’s protocols. All RT reactions, including no-template controls and RT minus controls, were run in triplicate in GeneAmp PCR 9700 Thermocycler (Applied Biosystems). All the primers used were listed in supplementary material 1, Table S2.

### Statistical analyses

Data are presented as mean ± SEM, and analyzed using GraphPad Prism 5 statistical analysis and graphing software. Unpaired student t test was used to determine differences between the two groups. Multiple group comparisons were performed using one-way ANOVA with the post hoc test of Tukey's. P < 0.05 was considered significant.

## Results

### 3-DZNeP alleviates the lung injury and prevents subsequent fibrosis in LPS-induced ALI/ARDS model

**Fig. 1.**
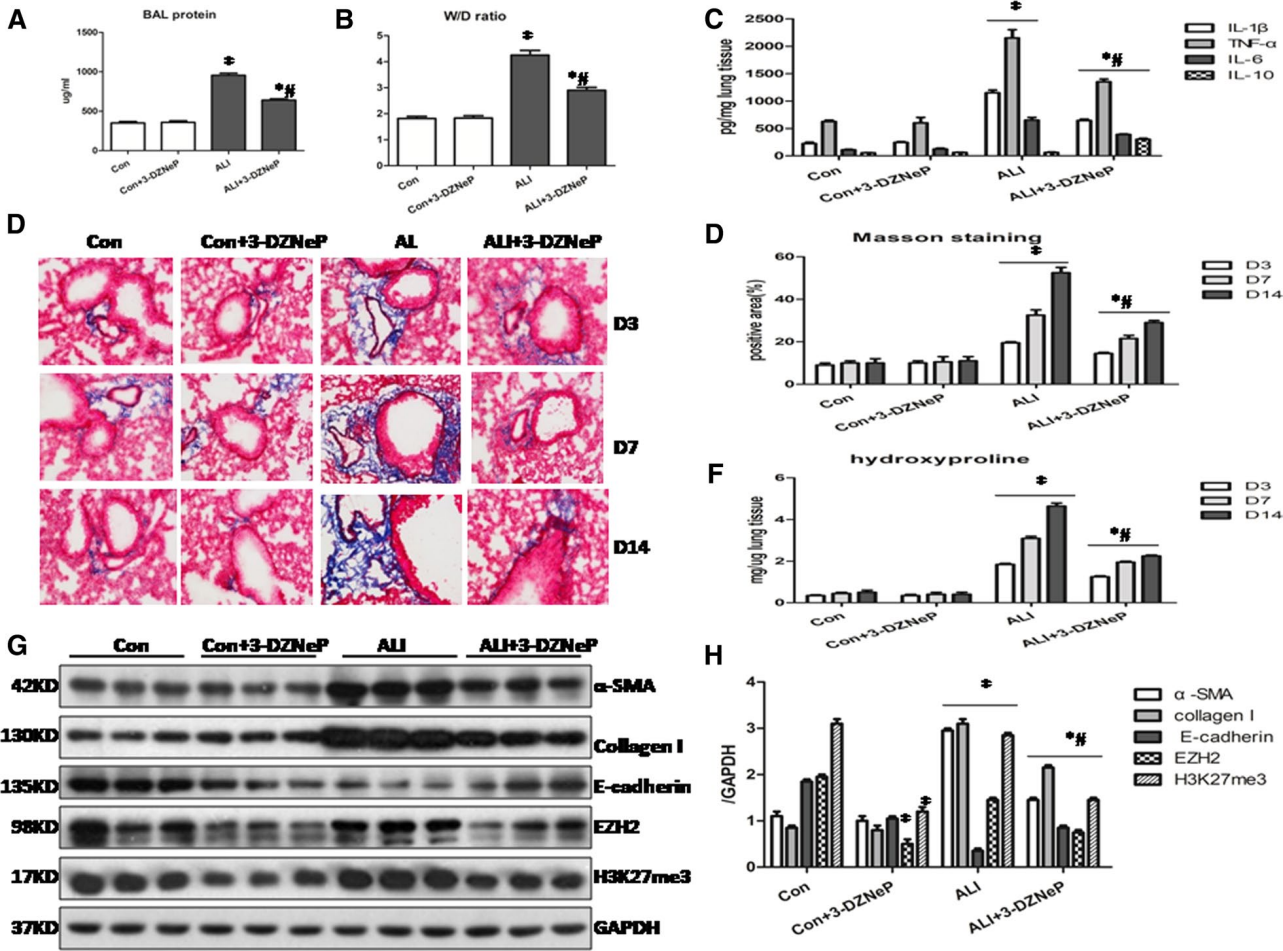
3-DZNeP ameliorates the lung injury and represses subsequent fibrosis in ALI/ARDS animal model. **A** Quantification of protein levels in the bronchial alveolar lavage (BAL) on Day 1 after intratracheal LPS in the control, control + 3-DZNeP, ALI, ALI + 3-DZNeP groups. **B** Evaluation of wet/dry weight ratio is compared among control, control + 3-DZNeP, ALI, ALI + 3-DZNeP groups. **C** Quantification of IL-1β, IL-6, TNF-a and IL-10 levels in the lung homogenates in the control, control + 3-DZNeP, ALI, ALI + 3-DZNeP groups. **D** Photomicrographs illustrate Masson trichrome staining to highlight collagen deposition on Day 3, Day 7 and Day 14 after treatments as indicated (n ≥ 3 in each group; images are representative examples, ×400 magnification). **E** The Masson trichrome–positive interstitial area (blue) relative to the whole area from 10 random fields was analyzed. **F** Quantification of hydroxyproline on Day 3, Day 7 and Day 14 after intratracheal LPS in different groups. **G** Lung tissue lysates were subjected to immunoblot analysis with specific antibodies against α-SMA, Collagen I, E-cadherin, EZH2, H3K27me3 and GAPDH. **F** Expression levels of indicated proteins were quantified by densitometry and normalized with GAPDH. All data are expressed as mean ± SEM. (n = 9–15/group, *P < 0.05 vs. control and 3-DZNeP group, ^#^P < 0.05 vs. ALI group, determined by one-way ANOVA for multiple group comparisons)

To validate the effects of EZH2 inhibitor on lung injury and fibroproliferation in vivo, we utilized a LPS-induced mouse ALI/ARDS model. Compared with the vehicle treatment group, 3-DZNeP pretreated mice were protected from lung injury on Day 1 post LPS instillation as evidenced by reduced BAL protein and wet-to-dry weight ratio (W/D) (Fig. 1A, B). In parallel with our previous finding that 3-DZNeP significantly decreased the inflammatory cytokines in the lung homogenates of CLP-induced ALI mice [10], we noted a marked lower levels of IL-1β, TNF-α and IL-6, and higher levels of IL-10 (Fig. 1C) in the lung tissue on Day 1 post LPS instillation of 3-DZNeP-treated ALI mice comparing with untreated ALI mice and control mice.

Next, we set out to elucidate the role of EZH2 in mediating pulmonary fibrosis induced by LPS. In parallel with our previous report and others [2, 5, 6], we found a progressive fibroproliferation from Day 3 to a marked pulmonary fibrosis on Day 14 by mansion trichrome staining (Fig. 1D, E) as well as a high expression levels of hydroxyproline (Fig. 1F), a marker of collagen synthesis in the lungs of ALI mice. We also noted increased levels of α-smooth muscle actin (α-SMA) and collagen I while reduced expression of E-cadherin in the lung tissue of ALI mice comparing with control mice on Day 14 (Fig. 1G, H). It is noteworthy that 3-DZNeP treatment attenuated the fibrosis in the lung tissues as indicated by massion trichrome staining, lower levels of hydroxyproline and a reduction of markers of fibrosis as α-smooth muscle actin (α-SMA) and collagen I while reversal of epithelial marker protein as E-cadherin on Day 14 after LPS insult (Fig. 1E–H). Furthermore, we found an increased expression of EZH2 and H3K27me3 in the lung homogenates of the ALI mice on Day 14 comparing with the control mice (Fig. 1G, H). 3-DZNeP treatment significantly inhibited the levels of EZH2 and H3K27me3 in the lung tissue (Fig. 1G, H). Collectively, these data indicated that EZH2 inhibitor may repress the lung injury and progressive fibrosis in LPS-induced ALI/ARDS mice.

### Administration of 3-DZNeP protects against pulmonary fibrosis by inhibiting the M1 polarization while inducing M2-like macrophage differentiation

**Fig. 2.**
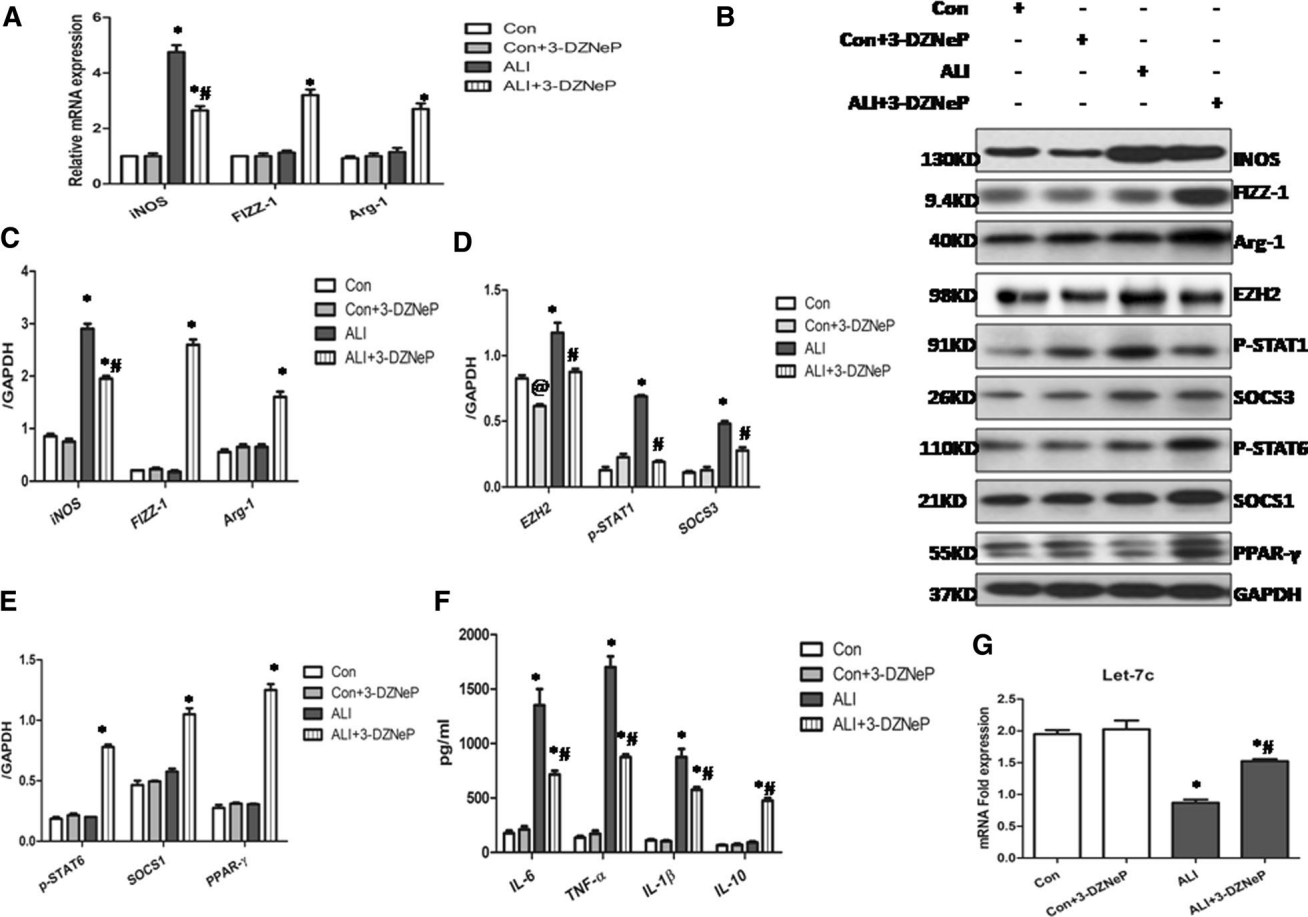
3-DZNeP inhibits M1 macrophage polarization while induces M2 macrophage differentiation in the BALF of LPS-induced pulmonary fibrosis. Monocytic cells were isolated from BALF on Day 14 after intratracheal LPS in the control, control + 3-DZNeP, ALI, ALI + 3-DZNeP groups. **A **The mRNA expression of M1 marker (iNOS) and M2 markers (Arg-1 and FIZZ-1) was measured by quantitative PCR. **B** Cell lysates were subjected to immunoblot analysis with specific antibodies against iNOS, FIZZ-1, Arg-1, EZH2, p-STAT1, SOCS3, p-STAT6, SOCS1, PPAR-γ and GAPDH. **C** Expression levels of iNOS, Arg-1 and FIZZ-1 were quantified by densitometry and normalized using GAPDH. **D** Expression levels of EZH2, p-STAT and SOCS3 were quantified by densitometry and normalized using GAPDH. **E** Expression levels of p-STAT6, SOCS1, PPAR-γ were quantified by densitometry and normalized using GAPDH. (F) The levels of IL-1β, IL-6, TNF-α and IL-10 were measured by ELISA in the BALF. **G** The expression level of let-7C was qualified by Real time PCR. All data are expressed as mean ± SEM. (n = 9–15/group, *P < 0.05 vs. control and 3-DZNeP group, ^#^P < 0.05 vs. ALI group, ^@^P < 0.05 vs. control group determined by one-way ANOVA for multiple group comparisons)

We and other investigators have found a pivotal role of M2-like macrophages in decreasing the fibroproliferative response after LPS-induced lung injury [5, 20, 21] and that EZH2 inhibition has the capacity to blunt M1 macrophage polarization in CLP-induced lung inflammation [10], therefore we next set out to examine the macrophage phenotypic changes in the LPS-induced pulmonary fibrosis by isolating monocytic cells from BALF on day 14 post LPS instillation and evaluating the mRNA and protein levels of macrophages markers. As shown in Fig. 2A–C, we observed a noted upregulated mRNA and protein expression of M1 marker inducible nitric oxide synthase (iNOS) while down-regulated levels of M2 marker (FIZZ-1 and Arg-1) in monocytes/macrophages sorted from BALF of fibrotic mice. Compared with the vehicle treatment ALI mice, the gene and protein levels of M1 maker (iNOS) and M1-asscocitaed cytokines including TNF-α, IL-1β and IL-6 were down-regulated on day 14 post-ALI in the 3-DZNeP treatment ALI mice, while M2-related markers (Arg-1 and FIZZ-1) and cytokines (IL-10) were noticed significantly up-regulated (Fig. 2A, C, F). Furthermore, we identified that p-STAT1 and SOCS3 were significantly inhibited while p-STAT6, SOCS1 and PPAR-γ were activated in the macrophages from BALF of 3-DZNeP treatment ALI mice comparing with vehicle treat ALI mice (Fig. 2B, D, E).

Previous studies have unraveled the suppressive effect of EZH2 on several miRNAs including let-7c in *ex-vivo* polarized macrophages [22, 23]; we further examined their expressions in the BALF of different treatment mice. We found that the protein levels of EZH2 were significantly upregulated in the sorted macrophages of ALI mice while downregulated with the administering of 3-DZNeP (Fig. 2B, D). Real time PCR further showed that the levels of let-7c were significantly decreased in the macrophages of vehicle treat mice comparing with PBS control mice, 3-DZNeP treatment can restore their expressions (Fig. 2G). Together, these data indicated that 3-DZNeP modulates pulmonary macrophages towards anti-inflammatory M2-like macrophages differentiation in the LPS-induced pulmonary fibrosis.

### Inhibition of EZH2 with 3-DZNeP or siRNA inhibits M1 macrophage polarization while promotes M2 phenotype in MH-S cells

To further explore the effect of 3-DZNeP on M1/M2 phenotypic shift of alveolar macrophages in the septic ALI/ARDS in vitro, we used LPS (50 ng/ml) and IL-4 (10 ng/ml) to stimulate MH-S cells toward M1 and M2 phenotype differentiation respectively. In a preliminary study, we examined the toxicity of 3-DZNeP in MH-S cells and revealed that the 3-DZNeP had no toxic effect and did not change cell viability, which was further indicated in the MTT assay (Supplementary material 2).

**Fig. 3.**
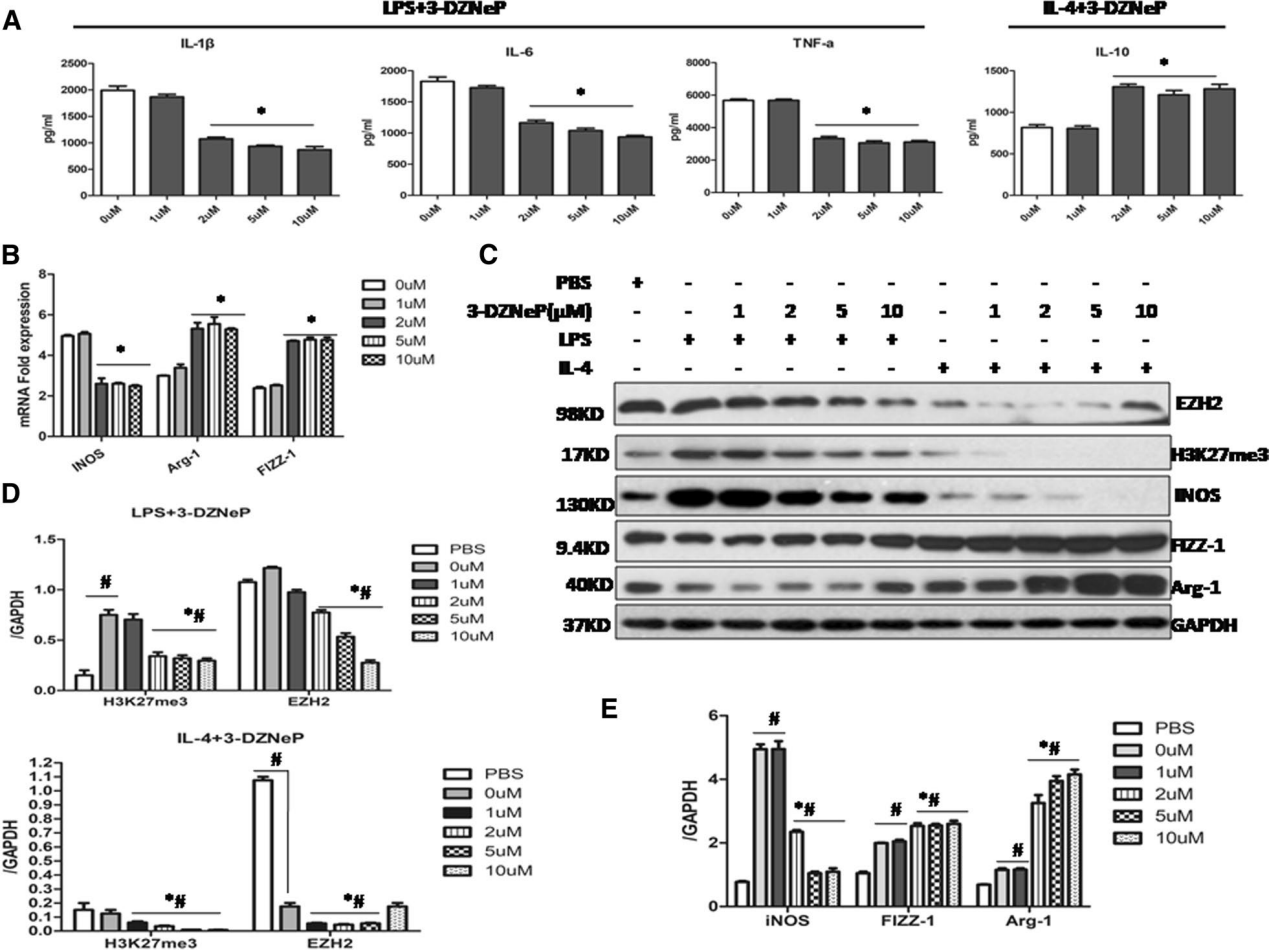
3-DZNeP suppressed M1 macrophage polarization while promoted M2 phenotype in MH-S cells. **A** The levels of IL-1β, IL-6, TNF-α were measured by ELISA in the supernatants of LPS stimulated MH-S cells and IL-10 levels were qualified by ELISA in IL-4 stimulated MH-S cells with different dose of 3-DZNeP (0, 1, 2, 5, 10 µM). **B** The mRNA expression of iNOS, Arg-1 and FIZZ-1 was measured by quantitative PCR. **C** Representative Western blot depicting MH-S cell lysates probed for EZH2, H3K27me3, iNOS, Arg-1, FIZZ-1 and GAPDH. **D** Expression levels of EZH2 and H3K27me3 were quantified by densitometry and normalized using GAPDH. **E** Expression levels of iNOS, Arg-1 and FIZZ-1 were quantified by densitometry and normalized using GAPDH. All data are expressed as mean ± SEM. (* *p* *<* 0.05 vs. MH-S cells with 0 µM 3-DZNeP, ^#^*p* *<* 0.05vs. MH-S cells with PBS determined by one-way ANOVA for multiple group comparisons)

To understand the effect of 3-DZNeP on molecular changes in ex vivo-induced macrophage subtypes, we cultured MH-S cells in the presence or absence of LPS (M1 polarization) or IL-4 (M2 phenotype) and treated them with different dosage of 3-DZNeP for 24 h. Firstly, we found that 3-DZNeP significantly decreased the expression of pro-inflammatory cytokines as IL-6, IL-1β and TNF-α in M1-polarized MH-S cells while increased IL-10 levels in M2 polarized states in a dose-dependent manner (Fig. 3A). Then, the qRT-PCR and western analysis for the M1 marker iNOS and the M2 marker Arg-1 and FIZZ-1 were performed. As shown in Fig. 3B-E, 3-DZNeP significantly inhibited the gene and protein expression of iNOS in the M1-polarized cells while promoted the expression of M2-specific marker genes including Arg-1 and FIZZ-1 in M2-polarization states. Moreover, the protein level of EZH2 and H3K27me3 was remarkably downregulated with the administration of 3-DZNeP (Fig. 3C, D).

**Fig. 4:**
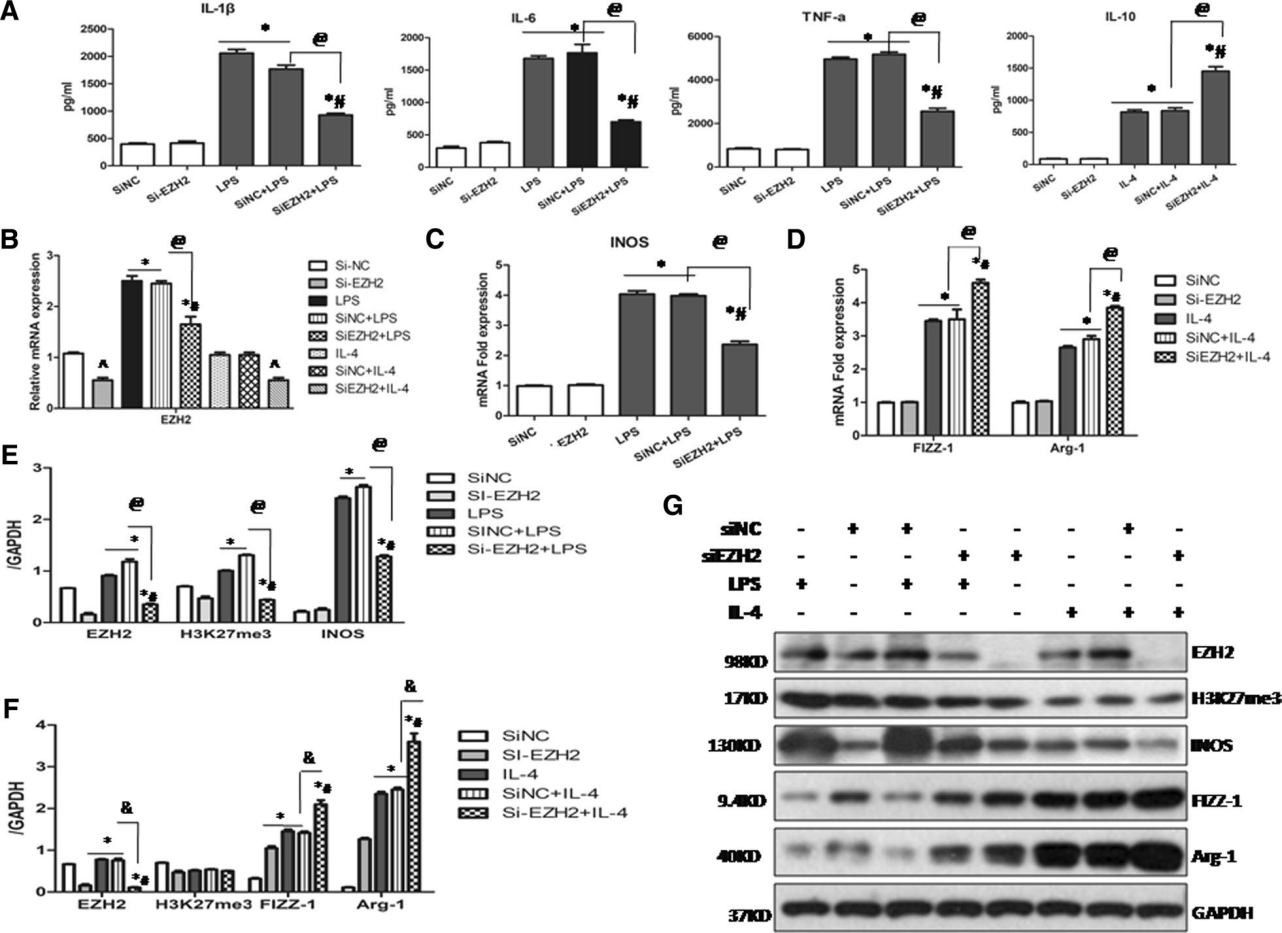
Knock down of EZH2 inhibited M1 macrophage polarization and promoted M2 phenotype in MH-S cells. MH-S cells were transfected with siRNA targeting EZH2 or scrambled siRNA and then exposed to either LPS (50 ng/ml) or IL-4 (10 ng/ml) for an additional 24 h. **A** The levels of IL-1β, IL-6, TNF-α were measured by ELISA in the supernatants of LPS stimulated MH-S cells and IL-10 levels were qualified by ELISA in IL-4 stimulated MH-S cells. **B** The mRNA expression of EZH2 was measured by quantitative PCR. **C** The mRNA expression of M1 marker (iNOS) was measured by quantitative PCR. **D** The mRNA expression of M2 marker (Arg-1 and FIZZ-1) was measured by quantitative PCR. **E** Expression levels of EZH2, H3K27me3 and iNOS were quantified by densitometry and normalized using GAPDH. **F** Expression levels of EZH2, H3K27me3, Arg-1 and FIZZ-1 were quantified by densitometry and normalized using GAPDH. **G** Representative Western blot depicting MH-S cell lysates probed for EZH2, H3K27me3, iNOS, Arg-1, FIZZ-1 and GAPDH. All data are expressed as mean ± SEM. (* *p* *<* 0.05 vs. SiNC and Si-EZH2 group, ^*#*^*p* *<* 0.05 vs. LPS or IL-4 stimulated group, ^@^*p* *<* 0.05 vs. SiNC + LPS group, & *p* *<* 0.05 vs. SiNC + IL-4 group and ^*p* *<* 0.05 vs. SiNC group determined by one-way ANOVA for multiple group comparisons)

Likewise, we further investigated the effect of genetic deletion of EZH2 with EZH2 siRNA on the two different polarization phenotypes in MH-S cells. Consistent with the above results, transfection of EZH2 siRNA also significantly inhibited the M1 polarization, as evidenced by down-regulation of IL-6, IL-1β, and TNF-α and M1 marker iNOS, and enhanced the M2 polarization, as indicated by up-regulation of IL-10 and M2 markers (Arg-1 and FIZZ-1) (Fig. 4A–G). Furthermore, we also noticed that the gene level of EZH2 and protein expressions of EZH2 and H3K27me3 were significantly inhibited with EZH2 siRNA (Fig. 4B, E–G). Taken together, these results indicate that genetic and pharmacological blockade of EZH2 suppressed LPS-induced M1 macrophage activation while promoted IL-4-induced M2 macrophage polarization in the cultured alveolar macrophage cell lines.

### 3-DZNeP or EZH2 siRNA suppressed STAT1 pathway in the M1-polarized MH-S cells while activated STAT6 pathway and PPAR-γ in the M2-marophage polarization

**Fig. 5:**
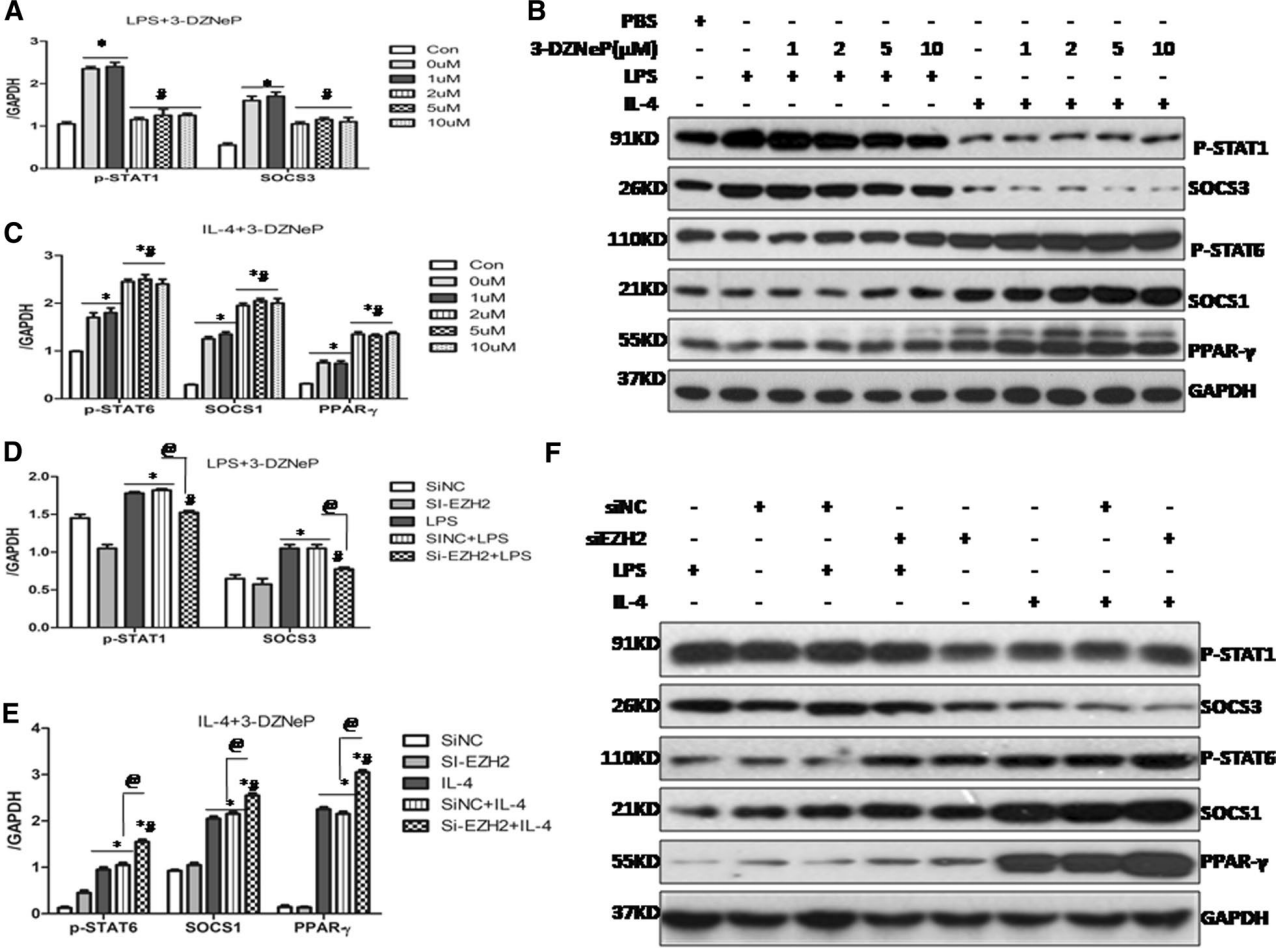
Blockade of EZH2 with 3-DZNeP or EZH2 siRNA inhibited STAT1 pathway in the M1-polarized MH-S cells while activated the STAT6 pathway and PPAR-γ in the M2-marophage polarization. **A **Expression levels of p-STAT1 and SOCS3 in the LPS-stimulated MH-S cells with different dosage of 3-DZNeP (0, 1, 2, 5, 10 µM) were quantified by densitometry and normalized using GAPDH. **B** Representative Western blot depicting either LPS or IL-4 stimulated MH-S cell lysates with different dose of 3-DZNeP (0, 1, 2, 5, 10 µM) probed for p-STA1, SOCS3, p-STAT6, SOCS1, PPAR-γ and GAPDH. **C** Expression levels of p-STAT6, SOCS1 and PPAR-γ in the IL-4 stimulated MH-S cells with different dosage of 3-DZNeP (0, 1, 2, 5, 10 µM) were quantified by densitometry and normalized using GAPDH. (* *p* *<* 0.05 vs. MH-S cells with 0  µM 3-DZNeP, ^#^*p* *<* 0.05vs. MH-S cells with PBS determined by one-way ANOVA for multiple group comparisons). **D** Expression levels of p-STAT1 and SOCS3 in the LPS-stimulated MH-S cells pretreatment with siRNA targeting EZH2 or scrambled siRNA were quantified by densitometry and normalized using GAPDH. **E** Expression levels of p-STAT6, SOCS1 and PPAR-γ in the LPS-stimulated MH-S cells pretreatment with siRNA targeting EZH2 or scrambled siRNA were quantified by densitometry and normalized using GAPDH. **F** Representative Western blot depicting either LPS or IL-4 stimulated MH-S cell lysates transfected with siRNA targeting EZH2 or scrambled siRNA, then probed for p-STA1, SOCS3, p-STAT6, SOCS1, PPAR-γ and GAPDH. All data are expressed as mean ± SEM. (*p *<* 0.05 vs. SiNC or Si-EZH2 group, ^*#*^*p* *<* 0.05 vs. LPS or IL-4 stimulated group, ^@^*p* *<* 0.05 vs. SiNC + IL-4 or SiNC + LPS group determined by one-way ANOVA for multiple group comparisons)

It is well known that the shift of macrophage phenotypes is regulated by several signaling pathways. JAK/STAT1 is the classical pathway involving in the M1 macrophage polarization, and SOCS3 is induced [4]. We recently documented that 3-DZNeP blunted M1 polarization in CLP-induced septic lung tissue in vivo partially through modulating the function of STAT1/SOCS3 [10]. As such, we hypothesized that 3-DZNeP or EZH2 siRNA would inhibit the M1 macrophage polarization in vitro by modulating the SOCS3/STAT1 pathway. As shown in Fig. 5A, B, the protein levels of phos-STAT1 (p-STAT1) were significantly up-regulated in the LPS-induced MH-S cells, 3-DZNeP inhibited the expression of p-STAT1 in a dose-dependent manner, which was duplicated in the EZH2 siRNA group (Fig. 5D, F). Furthermore, we noticed a significant up-regulated expression of SOCS3 in the M1-polaried MH-S cells, and that pretreatment with either 3-DZNeP or EZH2 siRNA down-regulated the levels of SOCS3 (Fig. 5A, B, D, F).

The shift from M1 to the M2 phenotype is regulated by several factors, and the SOCS1/STAT6 pathway is involved in this process [4]. Thus, we next set out to explore the effect of 3-DZNeP and Si-EZH2 on the activation of SOCS1/STAT6 in IL-4 induced M2-polarized MH-S cells. We observed that the protein level of p-STAT6 was significantly up-regulated in the M2-polarized cells, which was further augmented after pre-treatment of 3-DZNeP (Fig. 5B, C) or EZH2 siRNA (Fig. 5E, F). Moreover, SOCS1 was significantly activated in the in vitro polarized M2 macrophages which were further heightened after administering with either 3-DZNeP (Fig. 5B, C) or EZH2 siRNA (Fig. 5E, F). Previous studies have also suggested that the transcription factor PPAR-γ plays a pivotal role in the M1/M2 balance [18, 24]. As shown in Fig. 5B, C, E, F, our results showed that PPAR-γ was increased in the IL-4-stimulated cells and was further enhanced after 3-DZNeP or EZH2 siRNA treatment. Taken together, our results demonstrated that pharmacological or genetic blockade of EZH2 can inhibit STAT1 signaling pathway in M1-polarized MH-S cells, while stimulate the activation of STAT6 signaling and PPAR-γ in the M2-polarized alveolar macrophages in vitro.

### 3-DZNeP suppresses the in vivo epithelial to mesenchymal transition (EMT) by inhibiting activation of TGF-β/Smad signaling pathways in the ARDS-associated pulmonary fibrosis

**Fig. 6.**
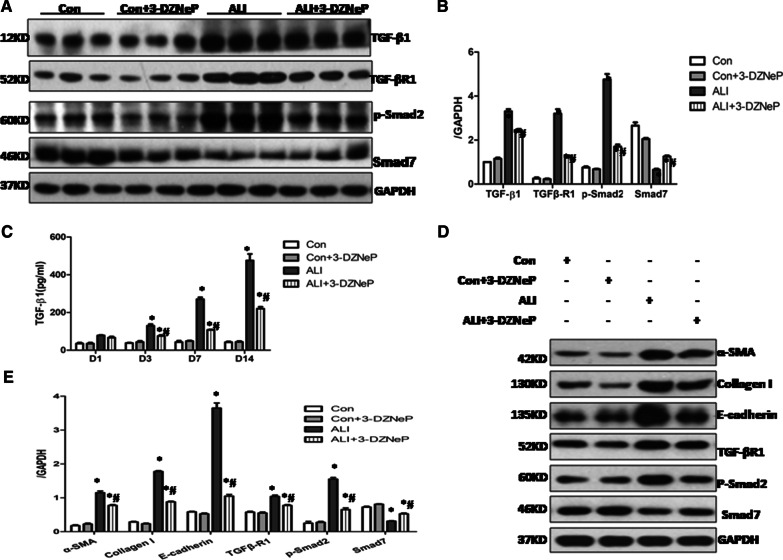
3-DZNeP lessens the in vivo and in *vitro* epithelial to mesenchymal transition (EMT) by inhibiting activation of TGF-β/Smad signaling pathways. **A** Lung tissue lysates on Day 14 after intratracheal LPS or PBS in the control, control + 3-DZNeP, ALI, ALI + 3-DZNeP groupswere subjected to immunoblot analysis with specific antibodies against TGF-β1, TGF-βR1, p-Smad2, Smad7 and GAPDH. **B** Expression levels of TGF-β1, TGF-βR1, p-Smad2 and Smad7were quantified by densitometry and normalized using GAPDH. **C** BALF was harvested on Day 1, Day 3, Day 7 and Day14 after intratracheal LPS in the control, control + 3-DZNeP, ALI, ALI + 3-DZNeP groups. The expression levels of TGF-β1 were measured by ELISA. All data are expressed as mean ± SEM. (n = 9–15/group, * P < 0.05 vs. control or 3-DZNeP group, #P < 0.05 vs. ALI group, determined by one-way ANOVA for multiple group comparisons). **D**, **E** Alveolar macrophages isolating from different treatment ALI mice on Day 14 after intratracheal LPS or PBS were co-cultured with mouse lung epithelial cell lines (MLE-12). Cell lysates were subjected to immunoblot analysis with specific antibodies against α-SMA, Collagen I, E-cadherin, TGF-βR1, p-Smad2, Smad7 and GAPDH (D).Expression levels of α-SMA, Collagen I, E-cadherin, TGF-βR1, p-Smad2, Smad7 were quantified by densitometry and normalized using GAPDH. All data are expressed as mean ± SEM. (n = 4–6/group, *P < 0.05 vs. control or 3-DZNeP group, ^#^P < 0.05 vs. ALI group, determined by one-way ANOVA for multiple group comparisons)

It is well known that EMT is involved in the pathological process related to LPS-induced pulmonary fibrosis [1, 2, 25]. As we have observed that 3-DZNeP can restore loss of E-cadherin and inhibit the excessive expression of α-SMA and Collagen I, two hallmarks of EMT and myofibroblasts, we further examined whether EZH2 plays a role in the regulation of the classical profibrotic TGF-β/Smad signaling pathways in LPS-induced ARDS-associated fibrosis. Western blotting results showed that expression levels of TGF-β1, TGF-βR1, p-Smad2 were increased in the fibrotic lung tissues whereas Smad-7 was reduced (Fig. 6A, B). Inhibition of EZH2 with 3-DZNeP suppressed the expression of TGF-β1, TGF-βR1 and p-Smad2, but partially preserved the levels of Smad-7 in the lung homogenates of ALI mice on Day 14 (Fig. 6A, B). These data suggest that 3-DZNeP may protect against EMT through inhibiting activation of the TGF-β/Smad pathway in the LPS-induced pulmonary fibrosis.

### Blockade of EZH2 with 3-DZNeP suppresses the in vitro EMT in co-cultured BALF and primary Lung epithelial cells through regulation of the TGF-β/Smad signaling

It has been reported that alveolar macrophages can interact with several other cell types such as epithelial, endothelial and immune cells during tissue repair and regeneration and fibrosis [26], however the underlying mechanism remains incompletely understood. Since we observed that the M2-like macrophages were elevated while EMT was suppressed in the alveolus of 3-DZNeP-treated fibrotic mice, we hypothesized that there exists a crosstalk between macrophages and epithelial cells in the alveolar microenvironment. To test this hypothesis, we first examined the dynamic changes of TGF-β1 over time in the BALF of the control (PBS and PBS + 3-DZNeP), vehicle and 3-DZNeP treat ALI mice. Figure 6C showed that levels of TGF-β1 began to rise from Day 3 and reached the peak on Day 14 in the vehicle group comparing with control mice, while a notable reduction of TGF-β1 was observed in the 3-DZNeP treatment group. Then we co-cultured alveolar macrophages obtained from different groups of mice on Day 14 with mouse lung epithelial cell lines (MLE-12) in vitro. As shown in Fig. 6D, E, the EMT was dramatically suppressed as shown by decreased expression of Collagen I and α-SMA while preserved expression of E-cadherin in the co-culture of MLE-12 with BALF from 3-DZNeP group comparing with that of the vehicle group. Then we further explored the potential effects of 3-DZNeP on the activation of TGF-β1 signaling pathway. In line with the in vivo results, there is a dramatic up-regulation of TGF-β1, TGF-βR1, p-Smad2 while down regulation of Smad7 in the vehicle-treatment group comparing with control group, while 3-DZNeP treatment resulted in the suppression of TGF-β1, TGF-βR1 and p-Smad2 and preservation of Smad7 in the co-culturing media (Fig. 6D, E). Therefore, it appears that 3-DZNeP can modulate the interaction between macrophages and epithelial cells and subsequently prevent EMT in the local microenvironment by inactivating TGF-β/Smad signaling pathway.

## Discussion

Pulmonary fibrosis remains the major cause of poor outcome in patients with ARDS, yet no targeted solutions exists so far. In the current study, we found that inhibition EZH2 with 3-DZNeP ameliorated lung inflammation and subsequent fibrosis in a model of LPS-induced ALI/ARDS mice. The underlying mechanism may be associated with promoting M2 polarization in the alveolar microenvironment and inhibiting the EMT by blocking TGF-β/Smad signaling pathway. Furthermore, we observed that pharmacological and genetic inhibition of EZH2 suppressed STAT1 pathway in the M1-polarized alveolar pulmonary cells and activated STAT6 pathway and PPAR-γ in the polarized M2-marophages*in vitro*. Thus, these data suggest that blockade of EZH2 may be a potential treatment option for prevention and alleviation of ARDS-associated fibroproliferation.

Pulmonary fibrosis develops concomitantly with severe ARDS which is marked by fibroblast proliferation with excessive deposition of matrix proteins [1, 2]. The balance between effective tissue repair and resolution of ARDS is a complicated process that is modulated by several immune cells [1, 2]. Macrophages are a predominant cell type in the alveolar air space and play a central role in the fibrotic and inflammatory response in ARDS but with paradoxical effects in both pulmonary fibrosis and healing process [3, 4]. Thus far, the underlying regulatory mechanisms to balance the positive and negative regulators on the profibrotic function of macrophages remain unclear. Our recent studies and other reports have documented that *ex-vivo* and *in-vivo* programmed M2 macrophages is positively involved in tissue repair and remodeling in ALI/ARDS with alleviation of lung injury, inflammation and later proliferation [5, 18, 21]. Macrophages maintain organ homeostasis and retain a functional dichotomy because of their presence in a dynamic microenvironment [4]. Recently, emerging evidence suggests that epigenetic mechanisms including DNA and histone modifications play an important role in modulating the shift of macrophage phenotypes [27]. Recently, we have also reported that pharmacological inhibition of EZH2 ameliorates the indirect lung injury and inflammation post sepsis through abrogating M1 macrophage polarization [10]. In the present study, we further confirmed that blockade of EZH2 with 3-DZNeP not only lessens the LPS-induced lung injury and inflammation but also prevents against pulmonary fibrosis. We identified an obvious shift of macrophages from M1 to M2 subtype, an anti-inflammatory phenotype in the BALF of 3-DZNeP treated mice. In vitro experiments further proved that pharmacological inhibition or genetic ablation of EZH2 could inhibit M1 polarization while promoting M2 phenotype switch in alveolar macrophage cell line. Large amount of studies have suggested a crucial role of miRNAs in regulating macrophage polarization in ARDS [22, 23, 28, 29]. In the current study, we found a lower expression of let-7c in the alveolar macrophages with a remarkable reservation after 3-DZNeP treatment, which is concordant with recent studies showing that let-7c suppresses polarization of macrophages to the M1 phenotype and enhances M2 polarization in sepsis [23], while pharmacologic and genetic inhibition of EZH2 could lead to let-7c-mediated macrophage inflammatory phenotype reversion via PAK1-dependent NF-kB signaling pathway [23]. Future investigations are still needed to elucidate the underlying molecular signals accounting for EZH2 induction and let-7c inhibition in the alveolar macrophages in response to LPS stimuli in mice. These findings have advanced our previous observation that EZH2-mediated epigenetic reprogramming contributes to macrophage polarization in the inflammatory cytokine response in sepsis, and further demonstrated that EZH2 inhibition could modulate the M1/M2 balance in favor of preventing the lung from LPS-induced fibrosis.

It is well established that M1 macrophage can be activated through activation of JAK/STAT1 signaling pathway, and that SOCS3 is upregulated [4]. However, whether the similar mechanism exists in the process of macrophage polarization in ARDS has not been explored. Our current studies demonstrated that pharmacological or genetic inhibition of EZH2 was able to inhibit the LPS-polarized M1 alveolar macrophage through modulating SOCS3/STAT1 pathway. Alternatively, several signaling pathways are involved in the phenotypic shift of M2 macrophages including SOCS1/STAT6 signaling pathway [4]. In our study, a significant upregulation of SOCS1 and activation of STAT6 were observed in the IL-4 stimulated alveolar macrophages with a heightened expression after treatment with 3-DZNeP or EZH2 siRNA. This is in line with another report showing that IL-4 can induce SOCS1 expression via regulating STAT6 signaling pathway [30]. PPARγ, a member of nuclear hormone receptor family, has also been documented to be involved in the process of M2 polarization [17, 20]. In the present study, we found that 3-DZNeP or EZH2 siRNA pretreatment dramatically increased PPAR-γ activity in the in vitro M2-polarizated alveolar macrophages. Moreover, we identified that the SOCS3/STAT1was inhibited while SOCS1/STAT6 and PPAR-γ were activated in the alveolar macrophages isolated from 3-DZNeP treated ALI mice. Therefore, our in vitro and in vivo experiments indicated that pharmacological or genetic inhibition of EZH2 inhibits the M1 macrophage polarization through SOCS3/STAT1 signaling pathway, while promotes the M2 subtypes by activating STAT6 pathway and PPAR-γ.

EMT is a process during which epithelial cells lose their epithelial cell marker such as E-cadherin and acquire mesenchymal characteristics as α-SMA. EMT has been confirmed to occur in several pathological processes, including tumor invasion [31], acute renal injury [32], and pulmonary fibrosis [33, 34]. Here, we demonstrated that the EMT also developed in the lung after LPS exposure, which was coincident with increased expression of EZH2 and H3K27me3; EZH2 inhibition significantly restored epithelial cellular markers and inhibited fibro-proliferation. This suggests that EZH2 is a critical mediator of EMT in LPS -induced lung fibrosis. It is well known that TGF-β1 is a key cytokine leading to EMT and TGF-β1 overexpression is associated with poorer prognosis in ARDS [25, 35].The downstream effects of TGF-β1 are mediated by Smad2/3, while Smad7 can bind TGF-βR1 and prevent TGF-β-associated Smad signaling [35]. It has been demonstrated that TGF-β1 induced EMT in alveolar epithelial cells both in vivo and in vitro via Smad2/3 activation [36, 37]. In parallel with these observations, we found that expression of TGF-β1, TGF-βR1, p-Smad2 were all upregulated in the fibrotic lung tissue while Smad7 was downregulated. In a model of bleomycin-induced idiopathic pulmonary fibrosis, it was reported that inhibition of EZH2 with 3-DZNeP had no effect on the levels of phosphorylated Smad2/3 but inhibited Smad2/3 nuclear translocation [11]. In contrast, we found that 3-DZNeP was effective in decreasing the levels of TGF-β1, TGF-βR1, p-Smad2 while preserving the expression of Smad7. The mechanism of 3-DZNeP-eliciated different effect in these studies remains unclear, but may be related to the use of different models. Nevertheless, apart from the Smad pathway, TGF-β1 induced EMT and fibrosis is also associated with activation of other signaling pathways, such as as MEK/ERK, PI3K/Akt, and Wnt/*β*-catenin [33]. Further investigations are needed to explore the relationships between EZH2 and Smad-independent pathways.

Although it is known that alveolar macrophages can interact with epithelial cells during the process of inflammation, resolution and tissue repair, the underlying mechanism remains incompletely understood in the alveolar micro-environment of ARDS [26].Our co-cultural system established using mouse epithelial cell line and BALF from ALI mice, allowed us to partially mimic a in vivo microenvironment. By using this system, we found that 3-DZNeP treatment dramatically reduced the expression of TGF-β1 in the BALF and inhibited the EMT. Furthermore, 3-DZNeP was effective in reducing the expression of TGF-βR1, p-Smad2 while preserving Smad7 expression in the ex-vivo culturing system. We had previously reported that transferring of M_2_ macrophages to the recipient’s lung could reduce the expression of TGF-β1 in the recipient’s lung [5], in this current study, we identified EZH2 inhibitor could modulate M2-like macrophage polarization in the BALF of ARDS mice, and further interact with epithelial cells and inhibit the EMT through the regulation of TGF-β1/Smad signaling pathway.

## Conclusions

In conclusion, we identified EZH2 as a key epigenetic regulator that promotes ARDS-associated fibrosis. We demonstrated for the first time that inhibition of EZH2 with 3-DZNeP or EZH2 siRNA attenuates lung injury and subsequent fibrosis by repressing the EMT through blocking TGF-β1/Smad signaling pathway and regulating shift of macrophage phenotypes by modulating the STAT/SOCS signaling pathway and PPAR-γ in vivo and in vitro. In addition, we revealed that the EZH2 inhibitor could regulate the crosstalk between alveolar macrophages and epithelial cells in favor of preventing EMT through TGF-β1/Smad signaling pathway. Since pharmacological EZH2 inhibitors have been used in the treatment of tumors in either preclinical studies or clinical trials [38, 39], our findings suggest the a potential use of EZH2 inhibitors as a treatment for ARDS-associated fibrosis.

## Data Availability

All data generated or analysed during this study are included in this published article [and its supplementary information files].

## Abbreviations

ARDS: Acute respiratory distress syndrome
α-SMA: α-Smooth muscle actin
BALF: Bronchoalveolar lavage fluid
CLP: Cecal ligation and puncture
EMT: Epithelial to mesenchymal transition
EZH2: Enhancer of zeste homolog 2
FBS: Fetal bovine serum
H3K27me3: Trimethylation of histone H3 at lysine 27
IL-1β: Interleukin-1β
iNOS: Inducible nitric oxide synthase
PBS: Phosphate buffered solution
PRC2: Polycomb-repressive complex 2
3-DZNeP: 3-Deazaneplanocin A
siRNA: Small interfering (si) RNA
TGF-β1: Transforming growth factor-β1
TNF-α: Tumor necrosis factor-α
